# Long Term Anticoagulation (4–16 Years) Stops Progression of Idiopathic Hip Osteonecrosis Associated with Familial Thrombophilia

**DOI:** 10.1155/2015/138382

**Published:** 2015-01-29

**Authors:** Charles J. Glueck, Richard A. Freiberg, Robert Wissman, Ping Wang

**Affiliations:** ^1^The Cholesterol, Metabolism, and Thrombosis Center, Jewish Hospital of Cincinnati, Cincinnati, OH 45207, USA; ^2^The Department of Orthopedic Surgery, Cincinnati VA Hospital, Cincinnati, OH 45207, USA; ^3^The Department of Radiology, College of Medicine, University of Cincinnati, Cincinnati, OH 45207, USA

## Abstract

In 6 patients with familial thrombophilia (5 Factor V (FV) Leiden heterozygotes, 1 with resistance to activated protein C (RAPC)), we prospectively assessed whether continuous longterm (4–16 years) anticoagulation would prevent progression of idiopathic osteonecrosis (ON), ameliorate pain, and facilitate functional recovery. Four men and 2 women (9 hips, 8 Ficat stage II, 1 stage I) were anticoagulated with enoxaparin (60 mg/day) for 3 months and subsequently with Coumadin, Xarelto, or Pradaxa, warranted by ≥2 prior thrombotic events. Anticoagulation was continued for 4, 4, 9, 13, 13, and 16 years, with serial clinical and X-ray follow-up. On 4–16-years anticoagulation, 9 hips in the 6 patients (8 originally Ficat II, 1 Ficat I) remained unchanged, contrasted to untreated ON Ficat stage II, where 50%–80% of hips progress to collapse (Ficat stages III-IV) within 2 years after diagnosis. Within 3, 3, 3, 9, and 16 months after starting anticoagulation, 5 patients became pain-free and remained asymptomatic throughout follow-up; the 6th patient required Percocet for pain. There were no significant bleeding episodes. Long term (4–16 years) anticoagulation initiated in Ficat stages I-II of idiopathic hip ON in patients with FV-RAPC changes the natural history of ON, stopping progression, resolving pain, and restoring function.

## 1. Introduction

The pathogenesis of osteonecrosis (ON) probably reflects a “multiple etiology” [1] model. We [2] and then others [3] have postulated a sequence for development of ON: venous thrombosis due to thrombophilia-hypofibrinolysis [4] causes osseous venous outflow obstruction, leading to increased intraosseous venous pressure, reduced arterial flow, ischemia, and bone death. Experimental models of ON [5] confirm venous occlusion as a primary event.

Primary (idiopathic) ON of hips and knees [6] in adults [7] and Legg-Calve-Perthes disease in children [8] are commonly associated with heritable thrombophilia. Relationships have been described between ON and Factor V Leiden heterozygosity [9], hypofibrinolysis [10], or reduction of nitric oxide (NO) production by the T-786C mutation of the endothelial nitric oxide synthase gene (eNOS) [10]. The association of heritable thrombophilia-hypofibrinolysis with ON is important because the diagnosis provides an opportunity to decrease the frequency of total hip replacement (THR) [2]. We speculate that enoxaparin can stop the progression of Ficat stages I and II primary ON of the femoral head by facilitating lysis of intraosseous thrombi, allowing bone healing [11]. Moreover, thrombophilic patients recognized before THR should be candidates for increased intensity and duration of postoperative anticoagulation [12] because of increased risk of postoperative thromboembolic disease.

Previously, in a prospective study, we hypothesized that an FDA-limited 3-month course of enoxaparin (60 mg/day) [11] would prevent progression of Ficat stage I-II primary ON of hip(s) associated with thrombophilia-hypofibrinolysis. After 3 months on enoxaparin, 20 patients (30 hips) with thrombophilia-hypofibrinolysis and Ficat stage I-II primary ON of ≥1 hip were followed for 4 to 7 years [2]. Maintenance of Ficat stages I-II versus progression to stages III-IV or THR over a follow-up period of 4 to 7 years was the study's endpoint. The first 16 patients (25 hips) received enoxaparin (60 mg/day) for 3 months [2], mandated by an FDA approved protocol [11]. The next 4 patients (5 hips) received enoxaparin 1.5 mg/kg/day for 3 months [2]. Based on intent to treat, at 4-year follow-up, 24 of the original 30 hips (80%) remained unchanged (Ficat stage I or II), as did 18 of 30 hips (60%) in 7 years [2]. Compared with untreated historical controls (approximately 20%–50% 2-year hip survival) [13], 4-year survival of 80% of hips, based on intent to treat, suggests that the original 12-week enoxaparin treatment [11] produces lasting benefit [2] in primary ON patients with heritable thrombophilia-hypofibrinolysis. Recently, in a retrospective study, Chotanaphuti et al. [14] studied 36 patients having at least 1 precollapse hip (Ficat stages I-II) who were anticoagulated for 12 weeks with 6000 units of enoxaparin daily. After 12 weeks on enoxaparin, 15 hips (58%) in the enoxaparin group and 5 hips of the control group (22%) remained of Ficat stage I or II, *P* = 0.042.

Based on our previous studies of short term anticoagulation (3 months of treatment with enoxaparin) [11] in patients with idiopathic ON and familial thrombophilia [2], our specific aim was to determine whether and to what degree continuous, long term anticoagulation in patients with Factor V Leiden heterozygosity or resistance to activated protein C (RAPC) would stop the progression of Ficat stage I or II idiopathic hip ON, ameliorate pain, and facilitate functional recovery. In the current report, we describe 5 patients heterozygous for the Factor V Leiden mutation and 1 with RAPC, with preanticoagulation Ficat stage I-II idiopathic ON of the hips (8: stage II, 1: stage I). All 6 patients received long term continuous anticoagulation because of ≥2 prior thrombotic events. We describe the success of continuous anticoagulation for 4 to 16 years, which stopped the progression of ON, ameliorated pain, and allowed resumption of full physical activities of daily living.

## 2. Methods

### 2.1. Study Design

The study followed a protocol approved by our institutional review board with signed informed consent.

In the order of their referral for diagnosis and therapy of ON, we assessed 535 patients for the Factor V Leiden mutation [9] and measurement of RAPC to identify both the common Gln506 mutation of the Factor V gene and the less common Arg306 mutation where the PCR for the Gln506 mutation is negative but RAPC is present, caused by the Arg306 mutation [15]. Both Factor V Leiden heterozygosity and RAPC represent the same type of thrombophilia, arising from different mutations of the Factor V gene.

Within ≤4 months after their initial diagnosis of ON, 5 patients with familial thrombophilia and ≥1 hip with Ficat stage I-II [1] idiopathic ON first participated in our FDA-limited 3-month treatment trial of enoxaparin (60 mg/day) in our outpatient clinical research center [11]. The 6th patient had developed multifocal ON (both knees, both shoulders, and right hip) 5 years earlier with bilateral total knee replacement (TKR) and bilateral total shoulder replacement, leaving her with a painful right hip, Ficat stage II. She was initially treated with enoxaparin 1.5 mg/kg/day in two divided doses for 90 days and then continued on Pradaxa 150 mg twice per day.

With long term anticoagulation warranted by ≥2 unprovoked thrombotic events, the 6 patients were then prospectively followed on chronic anticoagulation for 4, 4, 9, 13, 13, and 16 years (Table 1). In 4 of the 5 patients Coumadin was used in long term with the INR targeted between 2 and 3 and one patient was given Pradaxa 150 mg twice per day after 90 days of enoxaparin, while in 1 patient, Coumadin was later supplanted by Xarelto, 20 mg/day.

### 2.2. Participants in the Anticoagulant Study

The six patients, 2 women (1 African-American, 1 Caucasian) and 4 Caucasian males, were studied in the order of their referral to us for diagnosis and therapy of ON of the hips (Table 1). To enter our initial study [11] and its subsequent extension [2], patients were required to have idiopathic ON with ≥1 hip staged as Ficat I or II [1] and familial thrombophilia-hypofibrinolysis, assessed by previously published polymerase chain reaction (PCR) [16, 17] and serologic [17–20] measures. Five patients participated in our original enoxaparin trial of 20 patients [11] and the 6th one participated in our extended enoxaparin trial; all 6 were taken from a cohort of 535 patients with ON of the hips serially evaluated in our center in the order of their referral, all having measures of thrombophilia and hypofibrinolysis.

PA and frog leg lateral X-rays were taken at study entry, 3 and 6 months after initial enoxaparin therapy (60 mg/day) [11], and yearly thereafter. Initial MRI scans were utilized to verify the diagnosis of ON and were obtained at the end of follow-up for 5 of the 6 patients. ON was initially diagnosed and staged using the Ficat X-ray based classification [1] by a group of orthopedists and radiologists as previously described [11]. Assessment and staging [1] of X-rays in long term follow-up were done without concurrent knowledge of patients' symptoms or treatments.

Every 4 to 6 months, patients were interviewed to assess hip pain, restrictions on usual daily activity, and the need for analgesia medications.

## 3. Results

### 3.1. Total Cohort of 535 Patients with ON

Of the total cohort of 535 patients with ON, 63 were heterozygous for the V Leiden mutation (12%), and 20 (4%) had RAPC (V Leiden wild-type normal), altogether 83, 16% of the cohort, much more common than in 98 healthy normal controls (4.1%), *X* ^2^ = 9.1, *P* = 0.003.

### 3.2. Case Presentation: Six Idiopathic ON Patients with Long Term Anticoagulation

ON in our 6 long term anticoagulated patients was “idiopathic” that none had developed ON secondary to long term, high dose steroids, alcoholism, connective tissue disease, or antecedent traumatic fracture or dislocation [2, 11]. By selection for our initial enoxaparin trial [11] and its extension [2], all 6 patients had ≥1 hip with Ficat stage I and/or stage II osteonecrosis.

Five of the 6 patients (numbers 1, 2, 3, 5, and 6 (Table 1)) were found to be heterozygous for the Factor V Leiden mutation, and one (number 4, Table 1) had RAPC but wild-type normal V Leiden. None of these 6 patients had determinations of their familial thrombophilia before our initial [11] study. Because each patient had sustained 2 or more thrombotic events (including the ON) at entry to our initial enoxaparin study [11] or its extension, anticoagulation was continued with Coumadin (5 patients) or Pradaxa (1 patient) after completion of 3 months on enoxaparin [11]. In one patient (number 1), after 3 years on Coumadin, therapy was switched to Xarelto, 20 mg/day.

**Figure 1:**
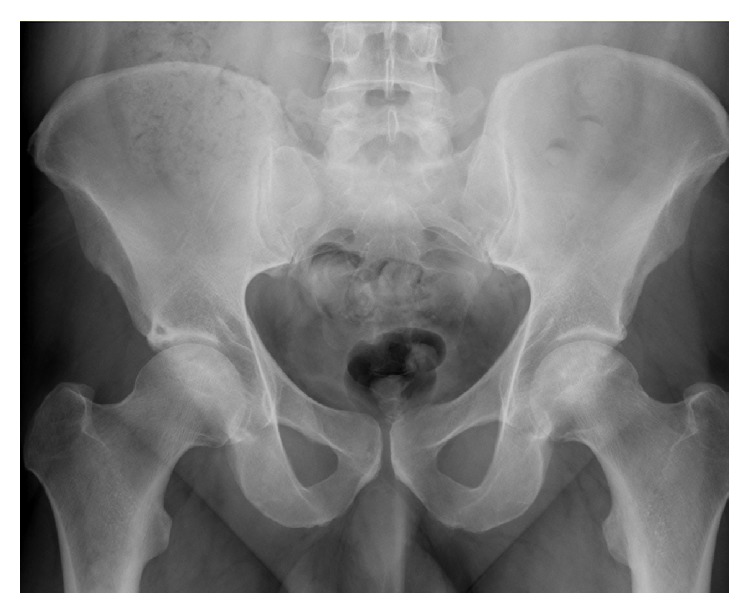
Anterior-posterior hip X-rays in patient number 3 at preanticoagulation study entry: both hips are Ficat stage II.

**Figure 2:**
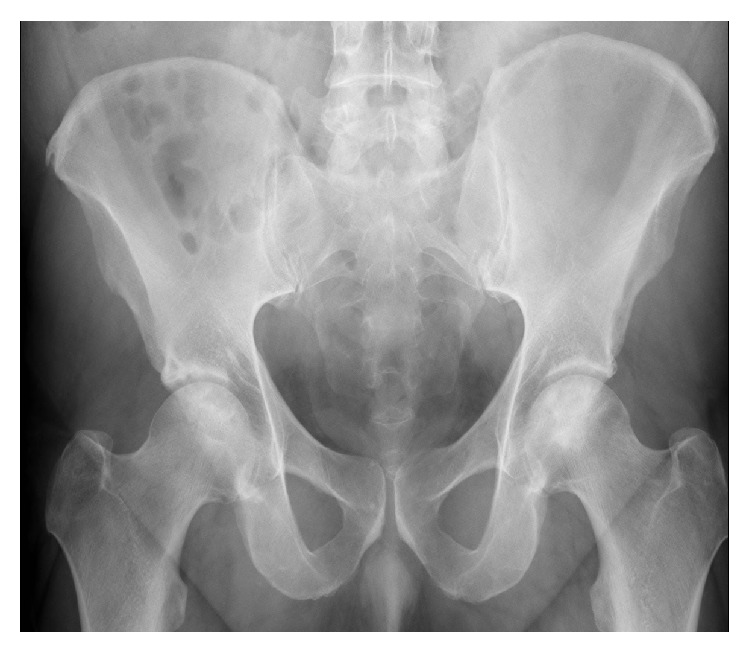
Anterior-posterior hip X-rays in patient number 3 after 9 years on Coumadin are unchanged, Ficat stage II.

**Figure 3:**
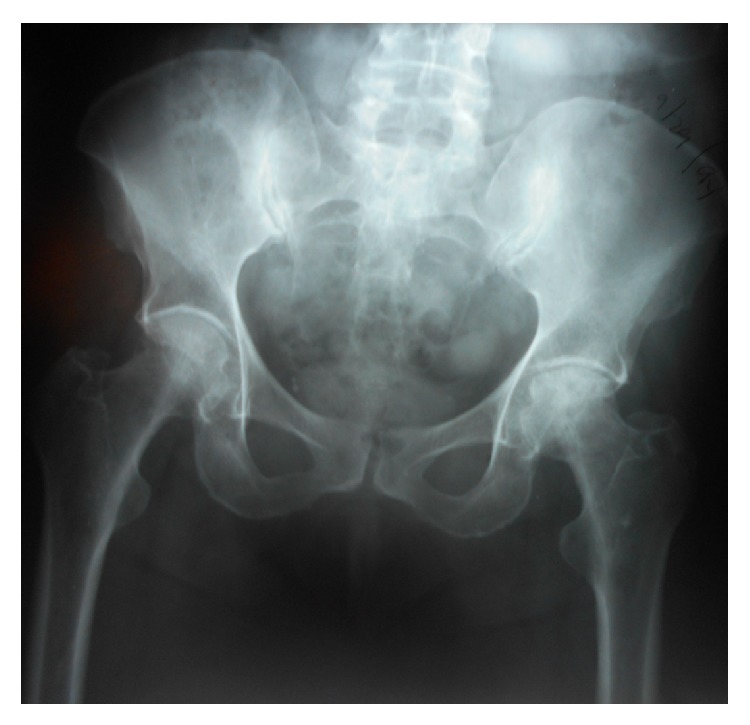
Anterior-posterior hip X-rays in patient number 4 at preanticoagulation study entry. The right hip is Ficat stage II and the left hip is Ficat stage III.

**Figure 4:**
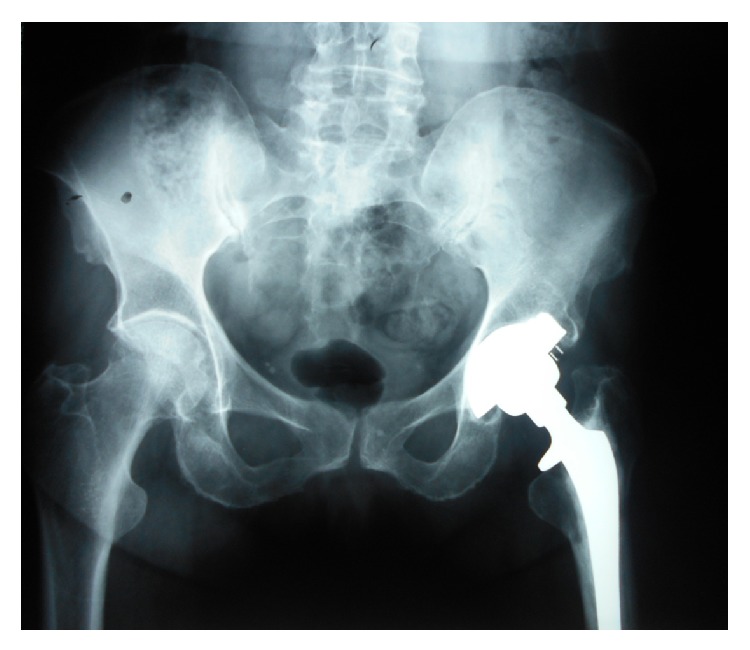
Anterior-posterior hip X-rays in patient number 4 after 5 years on Coumadin. The right hip is unchanged, Ficat stage II. The left hip, Ficat stage III at preanticoagulation study entry, was replaced before study entry.

**Figure 5:**
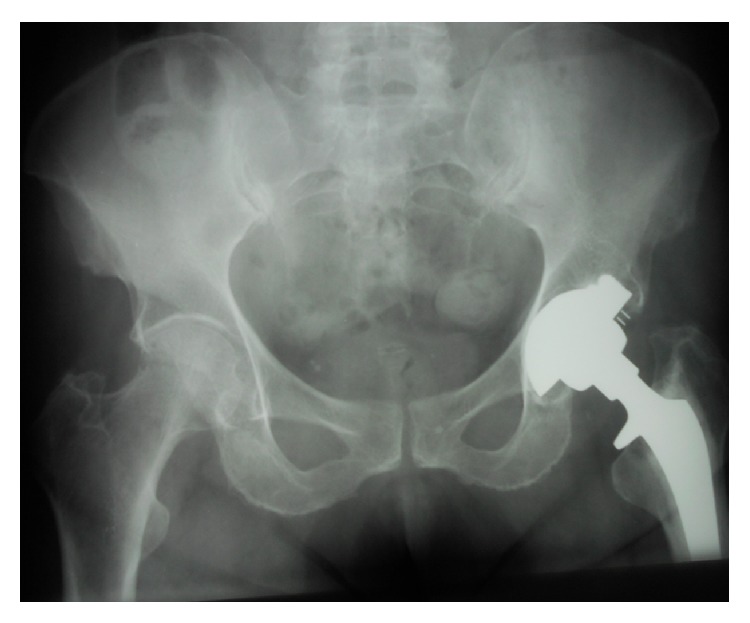
Anterior-posterior hip X-rays in patient number 4 after 13 years on Coumadin. The right hip is unchanged, Ficat stage II.

At follow-up of 4, 4, 9, 13, 13, and 16 years, respectively, Ficat staging remained unchanged from preanticoagulation study entry (Ficat stages I-II), with no hip collapse and no progression to osteoarthritis (Table 1). At 4-year follow-up in Cases numbers 1 and 2, preanticoagulation Ficat stages (I, II) were unchanged (Table 1). Comparing preanticoagulation entry and 9-year follow-up X-rays in Case number 3 revealed no change in Ficat stage II ON (Figures 1 and 2, Table 1). Comparing preanticoagulation entry and 5- and 13-year follow-up X-rays in Case number 4 (Figures 3, 4, and 5) revealed no change in Ficat stage II ON of the right hip in 5 and 13 years. The left hip, Ficat stage III at preanticoagulation entry (Figure 3), was replaced before entry into the anticoagulant trial (Figure 4).

Both hips in patient number 5, the right hip in patient number 6, and the right hip in patient number 2 became asymptomatic in 3 months (Table 1). Both hips in patient number 1 became asymptomatic in 9 months. In patient number 4, one hip was asymptomatic in 16 months (the other hip was replaced before the current study). Patient number 3 had persistent pain during physical activity requiring Percocet over 9-year follow-up (Table 1).

The 5 patients who became asymptomatic were able to carry out their usual daily activities and exercise (Table 1). At 4-year follow-up, patient number 1 plays golf 6 days per week and exercises at least 1 hour per day on an exercise bicycle. At 4-year follow-up, patient number 2 works full time lifting animals at her veterinary research center. At 13-year follow-up, patient number 4 does her housework, laundry, and gardening and walks 1 mile per day. At 13-year follow-up, patient number 5 actively rollerblades 1-2 hours per day or more (Table 1). At 16-year follow-up, patient number 6 works with heavy weight-lifting (up to 300 pounds) 1-2 hours per day, without symptoms (Table 1). Differing from the other 5 patients who required no analgesic agents after 3, 3, 3, 9, and 16 months (Table 1), at 9-year follow-up, patient number 3 often requires Percocet when physically active but has had no restriction of activities and has full range of motion (Table 1).

As displayed in Table 2, in the 5 patients having entry and end of follow-up MRIs, there were no changes in the MRIs, paralleling the finding of no change in the X-rays.

None of the patients sustained clinically significant bleeding during anticoagulation in the 4- to 16-year follow-up period.

## 4. Discussion

We have previously reported that enoxaparin (60 mg/day) given for 3 months to patients with Ficat stage I-II ON and thrombophilia-hypofibrinolysis can stop the progression of ON of the femoral head by facilitating lysis of intraosseous thrombi, allowing bone healing [2, 11]. After 2-month therapy with 60 mg enoxaparin, Chotanaphuti et al. [14] reported that 58% of patients with preanticoagulation ON (Ficat stages I-II) remained unchanged, versus 22% of control ON patients without enoxaparin, *P* = 0.042. Based on our previous studies of short term anticoagulation [11], we have speculated that continuous, long term anticoagulation in patients with Factor V Leiden heterozygosity or RAPC would stop the progression of Ficat stage I or II idiopathic ON and ameliorate hip pain.

The most common familial thrombophilia associated with ON [6, 7, 9] is the Factor V Leiden mutation and/or the closely related RAPC [15], found in 15.5% of 535 ON cases evaluated in our center.

Continuous anticoagulation for 4, 4, 9, 13, 13, and 16 years in 6 patients (9 hips), 5 heterozygous for the Factor V Leiden mutation and 1 with RAPC, stopped the progression of ON, documented by X-ray and MRI, and allowed 5 of the 6 patients to have pain-free, full activities and range of motion, while the 6th patient required Percocet for pain. This outcome is very favorable, particularly when compared to the natural history of untreated ON of the hips where untreated historical controls have been reported to have approximately 20%–40% 2-year hip survival [13, 21–24]. Mont et al. [23] have reported that “…untreated asymptomatic osteonecrosis has a high prevalence of progression to symptomatic disease and femoral head collapse.” Kang et al. [24] reported that symptoms developed in 38 of 68 patients with ON (55.9%) at a mean of 2.27 years after diagnosis and in 12 of 26 (46.2%) originally asymptomatic patients with idiopathic ON.

The major limitation of the current study was that it had a small number of subjects who had varying durations of treatment (4 to 16 years); however, 4 of the 6 patients had follow-up for 9 or more years. An optimal prospective study would be multicenter with thrombophilic subjects randomized to long term anticoagulation in one arm, anticoagulation plus core decompression with added stem cells in a second arm [25–28], and anticoagulation with core decompression [29] alone in a third arm.

Since our primary goal was to determine whether long term continuous anticoagulation would prevent collapse of the head of the femur (transition from Ficat stage I or II to stages III-IV) we used X-rays for Ficat [1] staging, since radiographs are highly specific for more advanced osteonecrosis (Ficat stage III or IV) but less sensitive for early changes (Ficat I) [30]. In the 5 of 6 patients in whom entry and follow-up MRIs were done, there were no changes in the percent of the femoral head affected with osteonecrosis, congruent with the X-ray findings.

## 5. Conclusions

If, as we and others have proposed, familial thrombophilia-hypofibrinolysis can cause ON [4, 6, 7, 10, 11, 31–37], then, as in the current study, long term anticoagulation started before segmental collapse of the head of the femur (Ficat stages I-II) in patients with thrombophilic Factor V Leiden or RAPC would be expected to stop the progression of idiopathic hip ON and relieve symptoms, thus preventing the need for total hip replacement.

## Figures and Tables

**Table 1:** Six patients, 9 hips (5 heterozygous for the V Leiden mutation, 1 with resistance to activated protein C). Anticoagulation for 4 to 16 years. Symptoms, nonprogression by X-ray, and Ficat stages at entry and on follow-up.

Patient	Race sex	Age at entry	Follow-up yrs	Symptom improvement on long term anticoagulation	Progression by X-ray	Ficat stages at entry and on follow-up
Number 1 (2 hips)	WM	56	4	Symptom-free hips in 9 months No restriction in activities Full range of motion	None in both hips	Ficat II, right hip, Ficat I, left hip, unchanged

Number 2 (right hip)	WF	29	4	Symptom-free right hip in 3 months No restriction in activities	None in right hip	Ficat II, right hip, unchanged

Number 3 (2 hips)	WM	48	9	Symptoms persistent in both hips requiring Percocet No restriction in activities Full range of motion	None in both hips	Ficat II, both hips, unchanged

Number 4 (right hip)	BF	69	13	Symptom-free right hip in 16 months No restriction in activities Full range of motion	None in right hip	Ficat II, right hip, unchanged

Number 5 (2 hips)	WM	49	13	Symptom-free hips in 3 months No restriction in activities Full range of motion including rollerblading	None in both hips	Ficat II, both hips, unchanged

Number 6 (right hip)	WM	32	16	Symptom-free right hip in 3 months No restriction in activities Full range of motion including weight-lifting	None in right hip	Ficat II, right hip, unchanged

**Table 2:** Six patients, 9 hips (5 heterozygous for the V Leiden mutation, 1 with resistance to activated protein C). Anticoagulation for 4–16 years. Entry MRI and nonprogression by MRI.

Patient	Follow-up (years)	MRI finding at pretreatment entry	Progression by follow-up MRI at end of study
Number 1 (2 hips)	4	Osteonecrosis of right femoral head involving 25% of the superior articular surface Small subcentimeter focus of low T1 and T2 signal intensity (sclerosis) in the left femoral head	None in both hips

Number 2 (right hip)	4	Subchondral osteonecrosis measures approximately 1 cm transversely by 2.2 cm in the AP dimension	None in right hip

Number 3 (2 hips)	9	No subchondral collapse, serpiginous lesion with low signal intensity, confirming Ficat stage II by X-ray	No repeat MRI done

Number 4 (right hip)	13	Osteonecrosis of right femoral head involving 20% of the superior articular surface	None in right hip

Number 5 (2 hips)	13	Osteonecrosis of right femoral head involving 30% of the superior articular surface Osteonecrosis of left femoral head involving 30% of the superior articular surface	None in both hips

Number 6 (right hip)	16	Osteonecrosis of right femoral head involving 30% of superior articular surface	None in right hip

## Abbreviations

ON: Osteonecrosis
THR: Total hip replacement
TKR: Total knee replacement
NO: Nitric oxide
eNOS: Endothelial nitric oxide synthase gene
RAPC: Resistance to activated protein C.
